# Biomarker-based clustering identifies distinct pulmonary function trajectories in early systemic sclerosis

**DOI:** 10.3389/fimmu.2026.1798420

**Published:** 2026-04-20

**Authors:** Saori Uesugi-Uchida, Yoshihide Asano, Hirahito Endo, Daisuke Goto, Masatoshi Jinnin, Yasushi Kawaguchi, Yuta Koike, Masataka Kuwana, Sumiaki Tanaka, Katsunari Makino, Takashi Matsushita, Sei-ichiro Motegi, Hayakazu Sumida, Ikuko Ueda-Hayakawa, Tadashi Toyama, Minoru Hasegawa

**Affiliations:** 1 Department of Dermatology, University of Fukui, Fukui, Japan; 2 Department of Dermatology, Tohoku University Graduate School of Medicine, Sendai, Japan; 3 Department of Rheumatology, Southern Tohoku General Hospital, Fukushima, Japan; 4 Division of Rheumatology, Ibaraki Prefectural Central Hospital and Cancer Center, Ibaraki, Japan; 5 Department of Dermatology, Wakayama Medical University Graduate School of Medicine, Wakayama, Japan; 6 Division of Rheumatology, Department of Internal Medicine, Tokyo Women’s Medical University, Tokyo, Japan; 7 Department of Dermatology, Nagasaki University Graduate School of Biomedical Sciences, Nagasaki, Japan; 8 Department of Allergy and Rheumatology, Nippon Medical School Graduate School of Medicine, Tokyo, Japan; 9 Department of Rheumatology, Kitasato University Medical Center, Kitamoto, Japan; 10 Department of Dermatology and Plastic Surgery, Faculty of Life Sciences, Kumamoto University, Kumamoto, Japan; 11 Department of Dermatology, Faculty of Medicine, Institute of Medical, Pharmaceutical and Health Sciences, Kanazawa University, Ishikawa, Japan; 12 Department of Dermatology, Gunma University Graduate School of Medicine, Gunma, Japan; 13 Department of Dermatology, The University of Tokyo Graduate School of Medicine, Tokyo, Japan; 14 Department of Dermatology, The University of Osaka Graduate School of Medicine, Suita, Japan; 15 Department of Nephrology, University of Fukui, Fukui, Japan

**Keywords:** adhesion molecule, biomarkers, chemokines, cluster analysis, cohort studies, interstitial lung disease, mixed-effects model, systemic sclerosis

## Abstract

**Background:**

Systemic sclerosis (SSc) is a heterogeneous autoimmune disease in which interstitial lung disease (ILD) is a major determinant of mortality. Given that certain chemokines and adhesion molecules may be involved in the inflammation, subsequent vascular injury, and fibrosis observed in SSc, their circulating levels in peripheral blood may reflect the disease processes ranging from inflammation to vascular damage and fibrotic remodeling. However, the potential of these biomarkers to identify patient subgroups with divergent pulmonary trajectories remains to be elucidated.

**Methods:**

We performed a retrospective analysis of prospectively collected data from patients with early severe SSc (diffuse cutaneous SSc irrespective of ILD status or limited cutaneous SSc with ILD; disease duration <5 years) who were enrolled in a multicenter cohort. Serum levels of five chemokines and four soluble adhesion molecules were quantified at baseline. Patients were classified based on these biomarker profiles using k-means clustering. Changes in pulmonary function were compared among clusters using relative changes in percent vital capacity (%VC).

**Results:**

Patients (n = 92) were classified into three clusters: Cluster 1 (n = 37) with elevated sICAM-1 and sE-selectin; Cluster 2 (n = 13) with elevated CCL2, CXCL8, and sP-selectin; and Cluster 3 (n = 42) with no distinctive biomarker pattern. Cluster 3 showed stable %VC and served as the reference group. Cluster 1 showed early decline (one-year difference: −8.41%; 95% CI: −12.62 to −4.20; *p* < 0.001) that attenuated by year two. In contrast, Cluster 2 showed progressive decline (two-year difference: −7.77%; 95% CI: −15.25 to −0.29; *p* = 0.042). These biomarker-defined patterns were consistent with a vasculopathic–fibrotic profile in Cluster 1 and an inflammatory–vascular profile in Cluster 2.

**Conclusion:**

Serum chemokine and adhesion molecule profiles may help stratify early severe SSc into biologically distinct subgroups with different pulmonary trajectories, supporting their potential utility for early risk stratification in SSc-ILD.

## Introduction

Systemic sclerosis (SSc) is a chronic autoimmune disease characterized by vascular injury, immune dysregulation, and progressive fibrosis of the skin and internal organs (1). Clinically, SSc is classified into limited cutaneous SSc (lcSSc), in which skin sclerosis is restricted to distal extremities and the face, and diffuse cutaneous SSc (dcSSc), which extends to the trunk and proximal limbs (2). Whereas lcSSc generally progresses slowly, dcSSc may show rapid progression of skin sclerosis or severe internal organ involvement within approximately three years after disease onset (3). Among the major complications, interstitial lung disease (ILD) is particularly important as it is the leading cause of mortality in SSc (4). Thus, early severe SSc—defined as dcSSc or lcSSc with ILD—represents a major clinical concern. Identifying patients at risk of ILD progression in the early disease stage is therefore essential to guide timely therapeutic intervention.

A broad range of circulating biomarkers—including disease-specific autoantibodies, cytokines, chemokines, and adhesion molecules—have been associated with SSc and SSc-ILD (5–7). In addition, Krebs von den Lungen-6 (KL-6), surfactant protein-D (SP-D), and CCL18 are recognized as markers of ILD severity and progression (8, 9). However, due to marked clinical heterogeneity and variable disease trajectories, no single biomarker has proven sufficient for risk stratification. While some studies have evaluated biomarker panels, their prognostic performance has not been consistently replicated across cohorts, and population differences further complicate their generalizability (10, 11).

Recent single-cell and pathological studies have highlighted inflammatory–vascular circuits as central to SSc pathogenesis, wherein activated endothelial cells and fibroblasts produce chemokines that recruit immune cells, while cell adhesion molecules mediate leukocyte trafficking (12). Because similar cellular processes and molecular signatures are observed in both skin and lung tissues in SSc, circulating chemokines and soluble adhesion molecules represent biologically plausible biomarkers linking cutaneous and pulmonary fibrosis.

We previously analyzed a Japanese multicenter cohort of early-stage SSc and identified clinical predictors of skin sclerosis and ILD (13–15). In the present study, we investigated the potential of baseline serum chemokine and adhesion molecule profiles to stratify patients according to subsequent pulmonary trajectories. To this end, we applied a clustering approach to classify patients with early severe SSc and compared longitudinal changes in pulmonary function among clusters.

## Materials and methods

### Study design and cohort

This study represents a retrospective analysis of data from a prospective multicenter cohort conducted between January 2002 and April 2013. Consecutive Japanese patients with early-stage SSc (disease duration <5 years) were enrolled from ten tertiary centers (Kanazawa University Hospital, Gunma University Hospital, Keio University Hospital, Kitasato University Hospital, Kumamoto University Hospital, Nagasaki University Hospital, The University of Tokyo Hospital, Toho University Omori Medical Center, Tokyo Women’s Medical University Hospital, and Tsukuba University Hospital). All patients fulfilled the 1980 American College of Rheumatology preliminary criteria for SSc (16) and were classified as either lcSSc or dcSSc (2).

For this analysis, we included only patients with early severe SSc, defined as dcSSc irrespective of ILD status or lcSSc accompanied by ILD, with disease duration <5 years from the first non-Raynaud’s symptom. Patients with infectious, malignant, or other inflammatory diseases were excluded.

Clinical data were collected annually during the cohort period. The study protocol was approved by the ethics committees of all participating centers, and written informed consent was obtained from all participants, including consent for the secondary use of data. Only patients who agreed to such secondary use were included in the present analysis. This analysis was additionally approved by the Research Ethics Committee of the University of Fukui (No. 20200116), and an opt-out procedure was implemented.

### Clinical assessment

Clinical variables included sex, age, modified Rodnan skin score (mRSS) (17), disease subtype, autoantibodies, digital ulcers, pitting scars, joint symptoms, ILD confirmed by high-resolution computed tomography (HRCT), estimated systolic pulmonary arterial pressure (sPAP) ≥ 35 mmHg by echocardiography, gastroesophageal reflux, erythrocyte sedimentation rate (ESR), percent predicted vital capacity (%VC), percent predicted diffusing capacity of the lung for carbon monoxide (%DLCO), Health Assessment Questionnaire Disability Index (HAQ-DI) (18), and use of immunosuppressants or vasodilators.

### Serum chemokines and adhesion molecules

Fresh venous blood samples were obtained from all registered patients at baseline. Serum levels of five chemokines (CCL2, CCL5, CXCL8, CXCL9, CXCL10) were measured using a cytometric bead array, and four soluble adhesion molecules (sICAM-1, sE-selectin, sL-selectin, sP-selectin) were measured using ELISA (14, 15). All serum samples were stored at −70 °C until analysis. The detection limits of each assay were as follows: CCL2 2.7 pg/mL, CCL5 1.0 pg/mL, CXCL8 0.2 pg/mL, CXCL9 2.5 pg/mL, CXCL10 2.8 pg/mL. sICAM-1 31.2 pg/mL, sE-selectin 93.8 pg/mL, sL-selectin 78.1 pg/mL, and sP-selectin 125 pg/ml.

### Statistical analysis

Cluster analysis was performed using the k-means method (19). The optimal number of clusters was determined using the Calinski–Harabasz index. Five chemokines and four adhesion molecule concentrations (as described above) were standardized and used as clustering variables. Pairwise comparisons of biomarker levels between clusters were performed using the Mann–Whitney U test. Given the small sample size, no adjustment for multiple comparisons was applied.

A linear mixed-effects model was used to compare changes in %VC among clusters, with relative change in %VC as the outcome, cluster groups, years since registration, and their interaction (year × cluster group) as fixed effects, patient-specific random intercepts and slopes as random effects, and baseline %VC, oral glucocorticoid use and cyclophosphamide pulse therapy during follow-up as covariates (20).

Similarly, a linear mixed-effects model was used to compare changes in %DLCO among clusters. Relative change in %DLCO was treated as the outcome variable, with cluster group, years since registration, and their interaction (year × cluster group) included as fixed effects. Patient-specific random intercepts and slopes were included as random effects, and baseline %DLCO, oral glucocorticoid use and cyclophosphamide pulse therapy during follow-up were included as covariates (21).

## Results

### Study population

**Figure 1:**
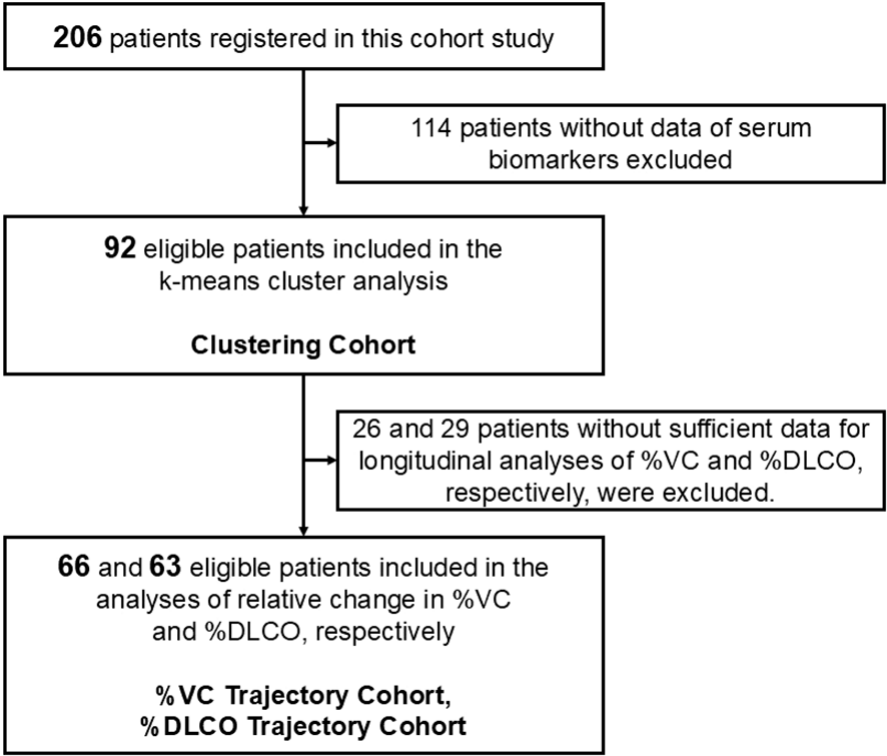
Identification of eligible patients. The flowchart shows the number of patients screened and included in the study.

A total of 206 patients were enrolled in the prospective cohort (**Figure 1**). Baseline serum chemokine and adhesion molecule data were available for 92 patients (hereafter referred to as the *Clustering Cohort*). Among these, 81 patients had ≥4 years of follow-up, 7 had 3 years, and 4 had 2 years. After excluding 26 patients without baseline %VC data, 66 patients were eligible for longitudinal analysis of pulmonary function (hereafter referred to as the *%VC Trajectory Cohort*). Similarly, after excluding 29 patients without baseline %DLCO data, 63 of the 92 patients were eligible for longitudinal analysis of %DLCO (*%DLCO Trajectory Cohort*).

### Cluster characteristics

The pseudo-F statistic was highest for the three-cluster solution (38.12), compared with the four-cluster (36.36) and five-cluster (33.58) solutions; therefore, three clusters were selected. Patients were classified into three clusters (Cluster 1, Cluster 2, and Cluster 3) based on baseline serum chemokine and adhesion molecule concentrations. These clusters were differentiated primarily by CCL2, CXCL8, sICAM-1, sE-selectin, and sP-selectin levels.

### Treatment exposure during follow-up

Baseline clinical characteristics stratified by cluster are shown in **Table 1**. Treatment exposure during follow-up is summarized in **Supplementary Tables 1**, **2**. Among patients in the clustering cohort, oral glucocorticoids were used in 79 cases (85.87%) and cyclophosphamide pulse therapy was administered in a limited number of patients(20 cases, 21.74%) during the 4-year follow-up period. In addition, one and five cases received oral tacrolimus and cyclosporin A, respectively, during follow-up. A total of 13 patients (14.13%) received neither glucocorticoids nor immunosuppressive agents during the 4-year follow-up period. No patients in this cohort received biologics or anti-fibrotic agents, reflecting the treatment standard during the original cohort period. In the adjusted linear mixed-effects model, additional adjustment for oral glucocorticoids and cyclophosphamide exposure during follow-up did not materially alter the association between cluster assignment and longitudinal %VC and %DLCO change.

**Table 1:** Baseline clinical characteristics by cluster.

Variable	All data (n=92)	Cluster 1 (n=37)	Cluster 2 (n=13)	Cluster 3 (n=42)
Gender female	65 (70.7)	24 (64.9)	8 (61.5)	33 (78.6)
Age, years	53 (37, 61)	54 (50, 62)	55 (53, 62)	49 (33, 59)
Duration of SSc, years	1 (1, 3)	1 (1, 3)	1 (1, 1)	2 (1, 3)
Subtype dcSSc	69 (75.0)	29 (78.4)	10 (76.9)	30 (71.4)
Subtype lcSSc	23 (25.0)	8 (21.6)	3 (23.1)	12 (28.6)
Anti-topoisomerase I Ab	56 (60.9)	26 (70.3)	7 (53.9)	23 (54.8)
Anticentromere Ab	12 (13.0)	5 (13.5)	1 (7.7)	6 (14.3)
Anti-U1 RNP Ab	20 (21.7)	4 (10.8)	4 (30.8)	12 (28.6)
MRSS	18 (10.5, 24)	19 (10, 23)	16 (11, 23)	15.5 (11, 24)
Joint contracture	23 (25.0)	9 (24.3)	5 (38.5)	9 (21.4)
Interstitial lung disease	54 (62.8) *	27 (75.0) †	9 (75.0) ‡	18 (47.4) §
Reflux/dysphagia symptoms	42 (45.7)	21 (56.8)	7 (53.9)	14 (33.3)
HAQ-DI	0.1 (0, 0.5)	0.3 (0, 0.6)	0.5 (0, 1.0)	0 (0, 0.1)

*n =86, †n=36, ‡n=12, §n=38.

n (%), median (25percentile, 75percentile).

dcSSc, diffuse cutaneous SSc; HAQ-DI, Health Assessment Questionnaire Disability Index; lcSSc, limited cutaneous SSc; mRSS, modified Rodnan total skin thickness score.

### Biomarker levels

**Figure 2:**
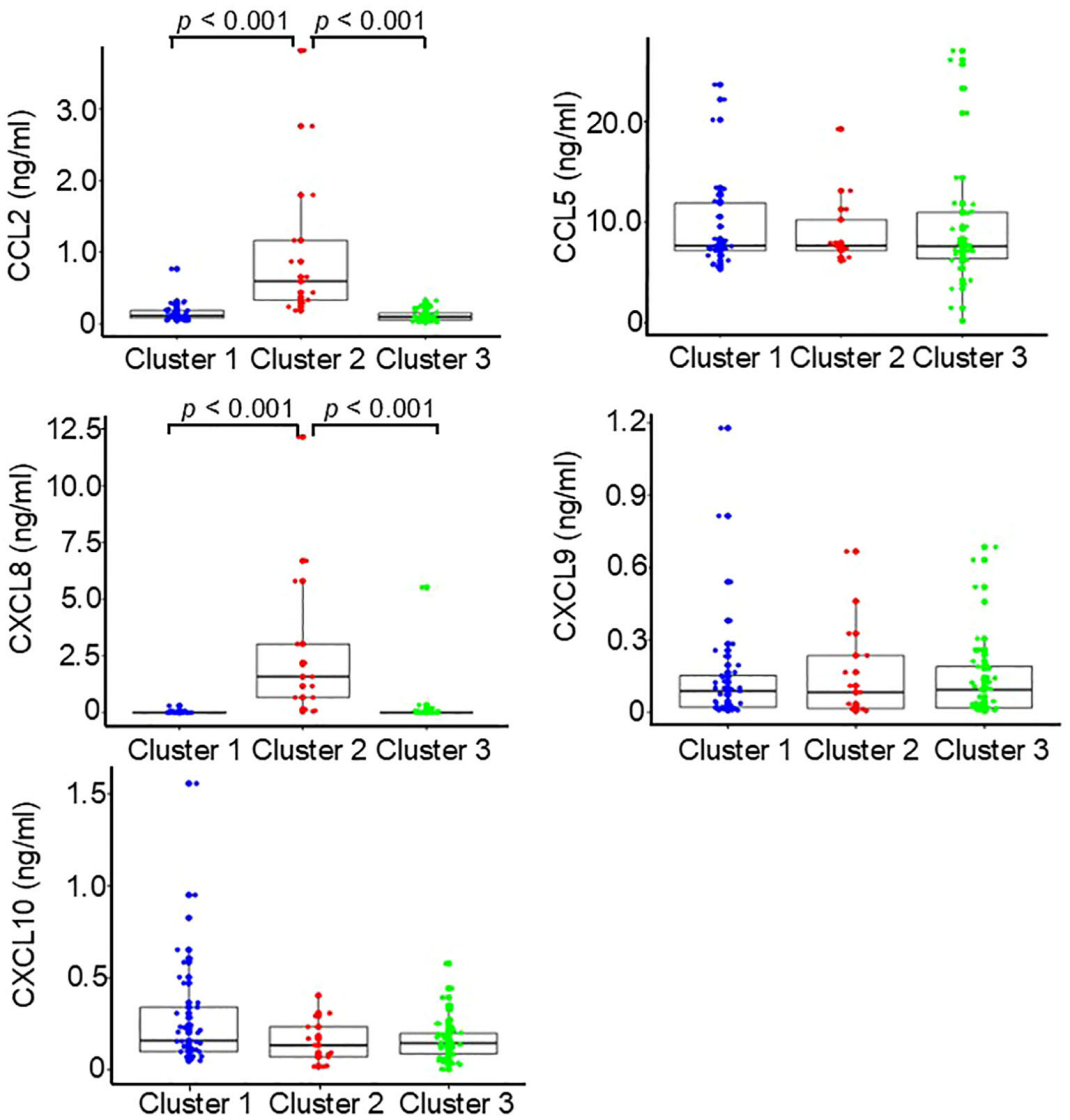
Baseline serum chemokines levels by cluster. Box-and-whisker plots show serum chemokine concentrations. Patients with systemic sclerosis (SSc) were stratified into three clusters using the k-means method based on baseline serum chemokine levels (Total n = 92; Cluster 1, n = 37; Cluster 2, n = 13; Cluster 3, n = 42).

**Figure 3:**
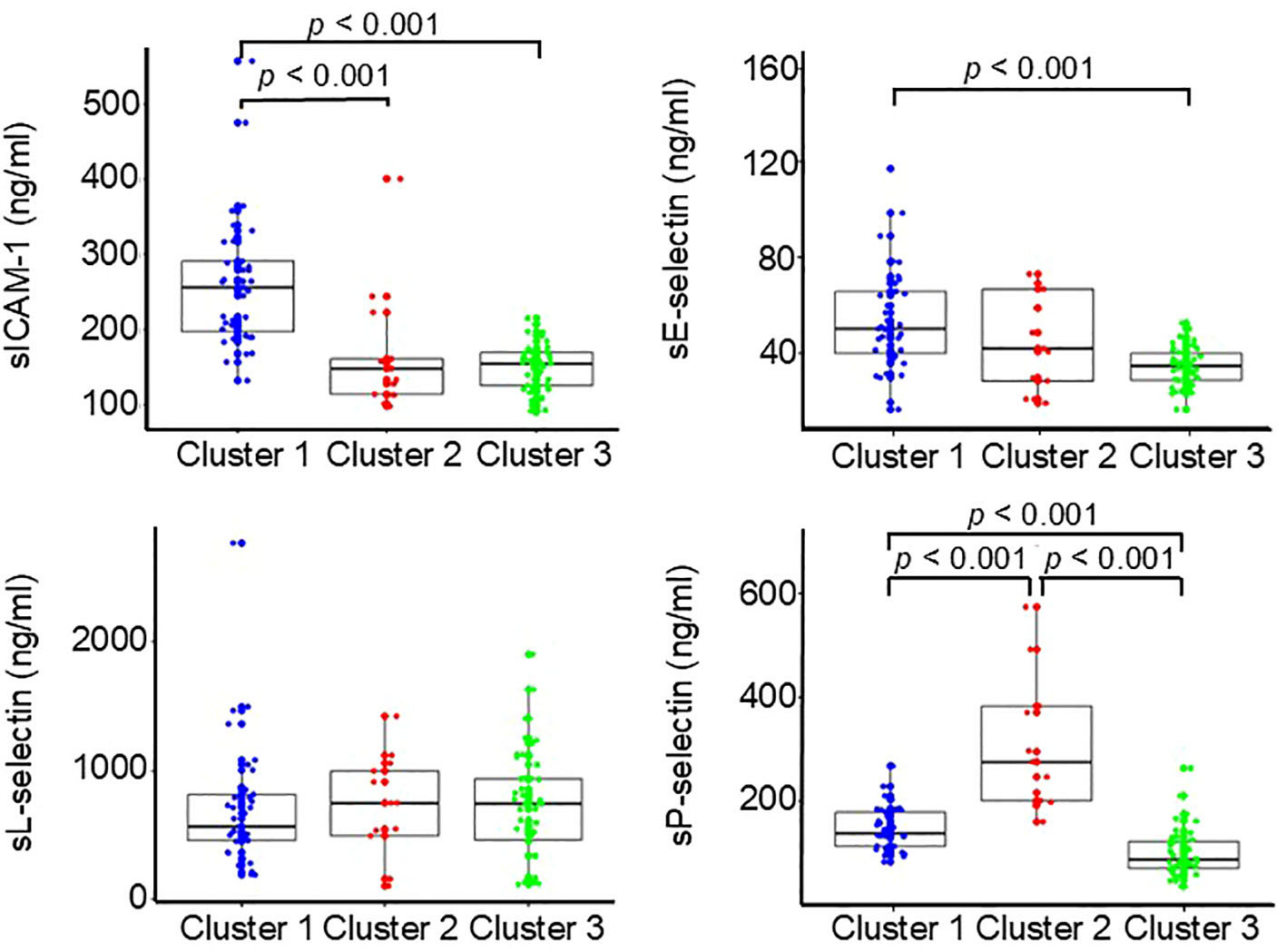
Baseline serum soluble adhesion molecules levels by cluster. Box-and-whisker plots show serum concentrations of soluble adhesion molecules in patients with SSc (Total n = 92; Cluster 1, n = 37; Cluster 2, n = 13; Cluster 3, n = 42).

Biomarker levels in each cluster are shown in **Figures 2** and **3**. Cluster 2 (n = 13) had higher serum CCL2, CXCL8, and sP-selectin levels than Clusters 1 (n = 37) and 3 (n = 42) (all *p* < 0.001). Cluster 1 had higher sICAM-1 than Clusters 2 and 3 (*p* < 0.001) and higher sE-selectin than Cluster 3 (*p* < 0.001). Cluster 3 showed no distinctive biomarker pattern. Given the limited number of patients in Cluster 2, these findings should be interpreted cautiously.

### Pulmonary function

**Figure 4:**
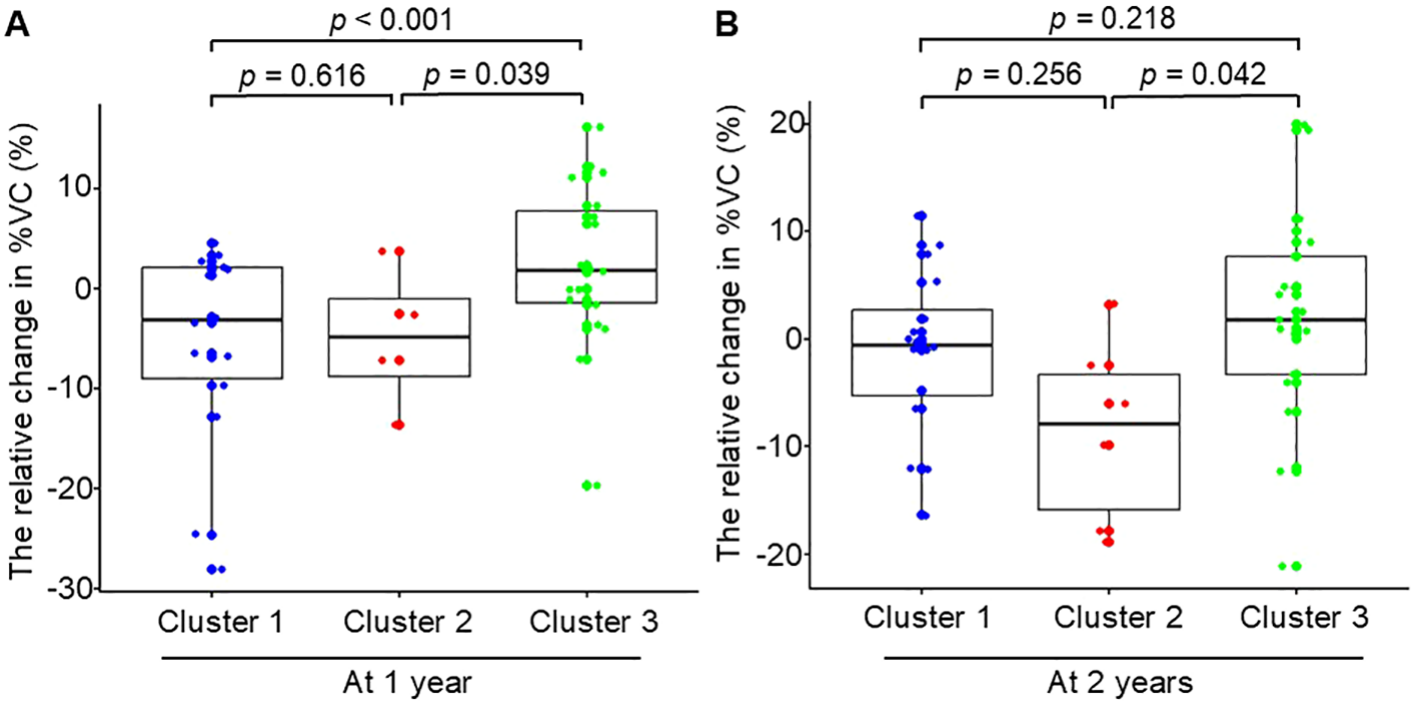
The relative change in %VC over two years by cluster. The relative change in %VC was calculated relative to baseline %VC at registration. The relative change in $\%VC=\frac{\%VC-baseline\;\%VC}{baseline\;\%VC}*100$. Box-and-whisker plots show the relative changes in %VC at **(A)** one year (Total n = 37; Cluster 1, n = 14; Cluster 2, n = 4; Cluster 3, n = 19) and at **(B)** two years (Total n = 43; Cluster 1, n = 16; Cluster 2, n = 6; Cluster 3, n = 21).

The clusters showed distinct patterns of %VC change over time. Cluster 3 showed stable %VC and was used as the reference. At 1 year, Cluster 1 showed a greater decline (difference: −8.41; 95% CI: −12.62 to −4.20; *p* < 0.001), as did Cluster 2 (difference: −6.72%; 95% CI: −13.11 to −0.33; *p* = 0.039) (**Figure 4A**). There was no significant differnece between Clusters 1 and 2 (*p* = 0.616). At 2 years, Cluster 1 no longer differed from Cluster 3. Cluster 2 continued to decline (difference: −7.77%; 95% CI: −15.25 to −0.29; *p* = 0.043) (**Figure 4B**). No difference was observed between Clusters 1 and 2 (*p* = 0.256). No differences were observed among clusters at 3 and 4 years (**Supplementary Figure 1**). Cluster 1 had significantly lower baseline absolute %VC compared with Cluster 3 (**Supplementary Figure 2**).

We additionally examined longitudinal changes in %DLCO. However, in contrast to %VC, longitudinal changes in %DLCO over 4 years did not differ significantly among clusters in the mixed-effects model (**Supplementary Figure 3**). At baseline, %DLCO tended to be lower in Cluster 1 compared with Clusters 3, although it was not significant (**Supplementary Figure 4**).

## Discussion

In this multicenter cohort of Japanese patients with early severe SSc, we classified patients into three clusters based on serum chemokine and adhesion molecule profiles and demonstrated that these clusters showed distinct short-term pulmonary trajectories. Notably, the clusters were not associated with changes in skin thickness, but were associated with distinct short-term changes in %VC, a clinically relevant surrogate for SSc-ILD severity. Cluster 3 showed no distinctive biomarker pattern, whereas Cluster 2 showed elevated CCL2, CXCL8, and sP-selectin, and Cluster 1 showed elevated sICAM-1 and sE-selectin.

Molecular studies support the biological relevance of these biomarker patterns. Single-cell and spatial transcriptomic analyses have demonstrated coordinated activation of endothelial, myeloid, and fibroblast populations in SSc tissues, highlighting chemokine-driven monocyte recruitment and reciprocal profibrotic signaling (12). Within this inflammatory–vascular network, CCL2 promotes monocyte trafficking and fibroblast activation (22), and elevated circulating CCL2 predicts ILD progression in early SSc (23). CXCL8 (IL-8) similarly amplifies neutrophil recruitment and its receptor CXCR2 has been explored as a therapeutic target in fibrosis models (24). Elevated CXCL8 in serum and bronchoalveolar lavage fluid (BALF) correlates with HRCT abnormalities and impaired pulmonary function, and has been suggested to be associated with an increased risk of mortality in SSc (25, 26). Furthermore, maladaptive CXCL8–CXCR1/2 signaling has been demonstrated in SSc neutrophils (27), reinforcing the link between CXCL8 biology and vascular inflammation. These data are consistent with the possibility that Cluster 2, characterized by elevated CCL2 and CXCL8, reflects an inflammatory–vascular profile associated with later pulmonary decline. Because Cluster 2 included a relatively small number of patients, these findings should be interpreted as hypothesis-generating and require confirmation in larger independent cohorts.

By contrast, soluble adhesion molecules integrate inflammatory signaling with cumulative endothelial injury. A recent meta-analysis confirmed that ICAM-1, E-selectin, and P-selectin are significantly elevated in SSc and serve as vascular biomarkers reflecting endothelial dysfunction (7). ICAM-1 is induced by pro-inflammatory cytokines such as TNF-α and IL-1β (28), and elevated sICAM-1 supports its potential utility as a screening biomarker for SSc-ILD (29). Combining sICAM-1 with SP-D and Ca15–3 also improves detection and risk stratification of ILD (30). E-selectin and P-selectin contribute to leukocyte rolling but differ in expression kinetics (31). Elevated soluble selectins correlate with reduced FVC and DLCO, and sE-selectin predicts pulmonary decline in SSc-ILD (29). Importantly, autologous hematopoietic stem cell transplantation improves nailfold capillaroscopic abnormalities and decreases dermal E-selectin expression, whereas circulating vascular markers remain elevated and are not fully normalized after transplantation (32). This observation suggests that circulating adhesion molecules may reflect persistent systemic endothelial activation even when local structural vascular improvement occurs. These findings parallel our Cluster 1 phenotype, characterized by elevated sICAM-1 and sE-selectin and lower baseline pulmonary function.

Taken together, chemokines such as CCL2 and CXCL8 may reflect “inflammatory–vascular” activity that precedes pulmonary function decline, while adhesion molecules such as ICAM-1 and E-selectin may reflect “vasculopathic–fibrotic” disease burden associated with baseline dysfunction. The complementary biology of Clusters 1 and 2 illustrates how integrating circulating biomarkers with clinical data can reveal distinct biologically meaningful SSc subgroups with prognostic value. Of note, inter-cluster differences diminished after year 3, suggesting that biomarker-informed endotyping may be most relevant in the early disease window when treatment decisions are most consequential.

In contrast to %VC, longitudinal %DLCO changes did not differ significantly among clusters, although baseline %DLCO tended to be lower in Cluster 1. Because DLCO reflects both alveolar–capillary and vascular function, this lower baseline value may indicate pre-existing endothelial injury, whereas %VC appears more sensitive to ongoing fibrotic progression.

This study has limitations. Although the underlying cohort was prospective, the present analysis was retrospective. Biomarker measurements relied on stored serum and treatment allocation was not controlled. Although oral glucocorticoid use was common across all clusters and cyclophosphamide pulse therapy was administered in a subset of patients, residual confounding related to treatment cannot be completely excluded despite covariate adjustment. In addition, the effects of cumulative treatment exposure and smoking may also have influenced pulmonary outcomes, but these data had not been systematically collected in the original cohort. Because of the small sample size, no adjustment for multiple comparisons was performed, and the biomarker analyses should therefore be considered exploratory. The *%VC Trajectory Cohort* included only patients with baseline %VC, potentially enriching for more severe ILD. Finally, other promising SSc-ILD biomarkers and quantitative imaging assessments using HRCT were not evaluated. Nevertheless, our findings extend prior work by demonstrating that baseline chemokine and adhesion molecule signatures stratify early severe SSc into biologically and clinically meaningful subgroups with distinct short-term pulmonary trajectories.

In conclusion, serum chemokine and adhesion molecule profiles can identify patient subgroups with distinct pulmonary trajectories in early severe SSc, supporting their potential for early risk stratification in SSc-ILD. Prospective validation in independent cohorts is warranted.

## Data Availability

The original contributions presented in the study are included in the article/**Supplementary Material**. Further inquiries can be directed to the corresponding author.

## Supplementary material

Supplementary Table 1 Proportion of patients receiving oral glucocorticoids during each observation interval.

Supplementary Table 2 Proportion of patients receiving cyclophosphamide pulse therapy during each observation interval.

Supplementary Figure 1 The relative changes in %VC after three and four years by cluster. Box-and-whisker plots show the relative changes in %VC at **(A)** three years (Total n = 42; Cluster 1, n = 17; Cluster 2, n = 4; Cluster 3, n = 21) and at **(B)** four years (Total n = 46; Cluster 1, n = 19; Cluster 2, n = 7; Cluster 3, n = 20).

Supplementary Figure 2 Baseline %VC in each cluster. Actual baseline %VC values for Clusters 1–3 are shown (Total n = 66; Cluster 1, n = 24; Cluster 2, n = 8; Cluster 3, n = 34).

Supplementary Figure 3 The relative changes in %DLCO over four years by cluster. The relative change in %DLCO was defined as compared to the baseline %DLCO at the time of registration. $The\;relative\;change\;in\;\%DLCO=\frac{\%DLCO-baseline\;\%DLCO}{baseline\;\%DLCO}*100$.Box-and-whisker plots show the relative changes in %DLCO from baseline (Total n = 63; Cluster 1, n = 26; Cluster 2, n = 7; Cluster 3, n = 30), at (A) one year (Total n = 39; Cluster 1, n = 15; Cluster 2, n = 6; Cluster 3, n = 18), at (B) two years (Total n = 39; Cluster 1, n = 18; Cluster 2, n = 6; Cluster 3, n = 15), at (C) three years (Total n = 44; Cluster 1, n = 19; Cluster 2, n = 5; Cluster 3, n = 20) and at (D) four years (Total n = 43; Cluster 1, n = 17; Cluster 2, n = 7; Cluster 3, n = 19).

Supplementary Figure 4 Baseline %DLCO in each cluster. Actual baseline %DLCO values for Clusters 1–3 are shown (Total n = 63; Cluster 1, n = 26; Cluster 2, n = 7; Cluster 3, n = 30).
