# Supplementary material for: Biomarker-based clustering identifies distinct pulmonary function trajectories in early systemic sclerosis
Source: Front Immunol. 2026 Apr 20;17:1798420. doi: 10.3389/fimmu.2026.1798420 (PMC13136265; doi:10.3389/fimmu.2026.1798420)
Supplement: Supplementary Table 1 — Proportion of patients receiving oral glucocorticoids during each observation interval. [file SupplementaryFile1.pdf]

Supplementary Table 1. Proportion of patients receiving oral glucocorticoids during each observation interval

	Baseline-1 year	1-2 years	2-3 years	3-4 years
Cluster 1 (n=37)	32 (86.5)	32 (86.5)	32 (86.5)	32 (86.5)
Cluster 2 (n=13)	11 (84.6)	12 (92.3)	13 (100)	13 (100)
Cluster 3 (n=42)	34 (81.0)	33 (78.6)	34 (81.0)	33 (78.6)

n (%)
