# Supplementary material for: Biomarker-based clustering identifies distinct pulmonary function trajectories in early systemic sclerosis
Source: Front Immunol. 2026 Apr 20;17:1798420. doi: 10.3389/fimmu.2026.1798420 (PMC13136265; doi:10.3389/fimmu.2026.1798420)
Supplement: Supplementary Table 2 — Proportion of patients receiving cyclophosphamide pulse therapy during each observation interval. [file SupplementaryFile2.pdf]

Supplementary Table 2. Proportion of patients receiving cyclophosphamide pulse therapy during each observation interval

	Baseline-1year	1-2 years	2-3 years	3-4 years
Cluster 1 (n=37)	12 (32.4)	6 (16.2)	7 (18.9)	8 (21.6)
Cluster 2 (n=13)	0 (0)	0 (0)	0 (0)	0 (0)
Cluster 3 (n=42)	2 (4.8)	3 (7.1)	3 (7.1)	4 (9.5)

n (%)
