# Supplementary figures and images for: Biomarker-based clustering identifies distinct pulmonary function trajectories in early systemic sclerosis
Source: Front Immunol. 2026 Apr 20;17:1798420. doi: 10.3389/fimmu.2026.1798420 (PMC13136265; doi:10.3389/fimmu.2026.1798420)

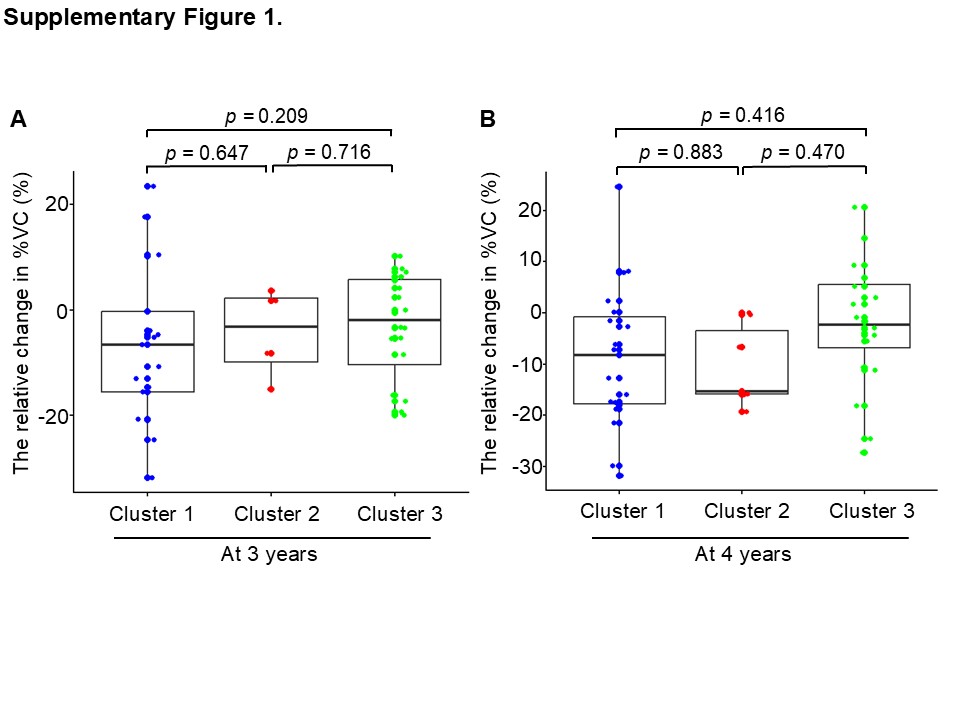

Supplement: Supplementary Figure 1 — The relative changes in %VC after three and four years by cluster. Box-and-whisker plots show the relative changes in %VC at (A) three years (Total n = 42; Cluster 1, n = 17; Cluster 2, n = 4; Cluster 3, n = 21) and at (B) four years (Total n = 46; Cluster 1, n = 19; Cluster 2, n = 7; Cluster 3, n = 20). [file Image1.jpeg]

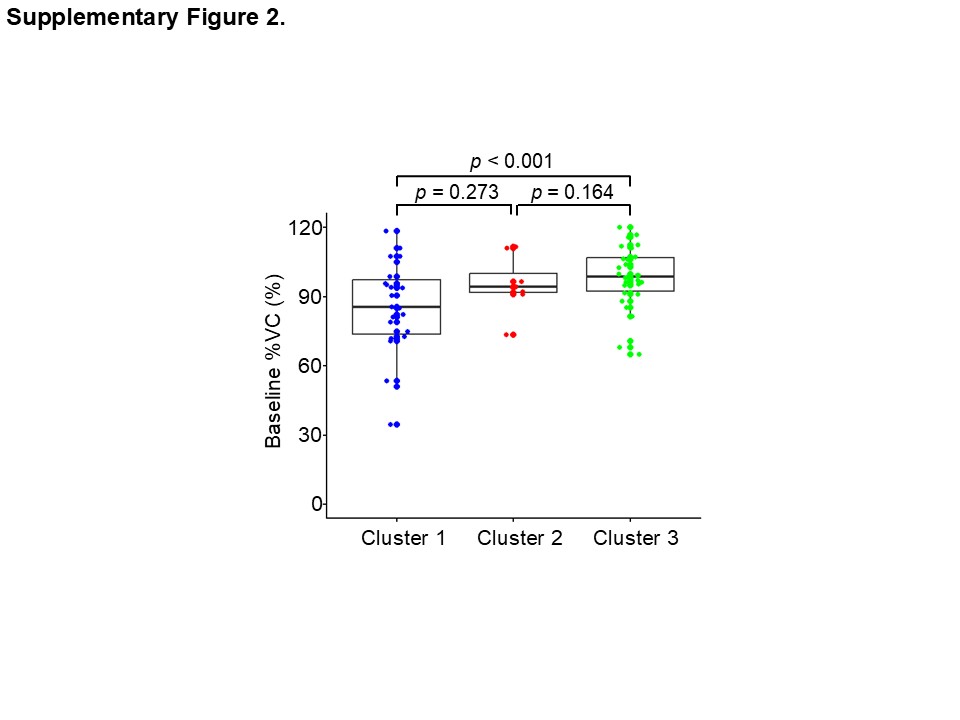

Supplement: Supplementary Figure 2 — Baseline %VC in each cluster. Actual baseline %VC values for Clusters 1–3 are shown (Total n = 66; Cluster 1, n = 24; Cluster 2, n = 8; Cluster 3, n = 34). [file Image2.jpeg]

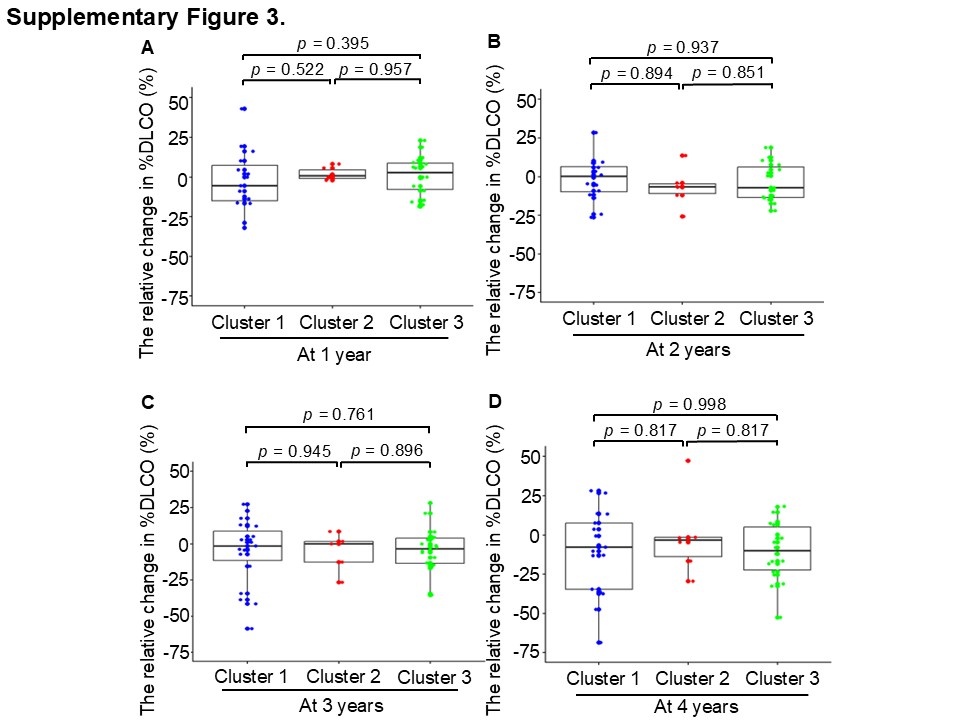

Supplement: Supplementary Figure 3 — The relative changes in %DLCO over four years by cluster. The relative change in %DLCO was defined as compared to the baseline %DLCO at the time of registration. The relative change in %DLCO=%DLCO−baseline %DLCObaseline %DLCO*100.Box-and-whisker plots show the relative changes in %DLCO from baseline (Total n = 63; Cluster 1, n = 26; Cluster 2, n = 7; Cluster 3, n = 30), at (A) one year (Total n = 39; Cluster 1, n = 15; Cluster 2, n = 6; Cluster 3, n = 18), at (B) two years (Total n = 39; Cluster 1, n = 18; Cluster 2, n = 6; Cluster 3, n = 15), at (C) three years (Total n = 44; Cluster 1, n = 19; Cluster 2, n = 5; Cluster 3, n = 20) and at (D) four years (Total n = 43; Cluster 1, n = 17; Cluster 2, n = 7; Cluster 3, n = 19). [file Image3.jpeg]

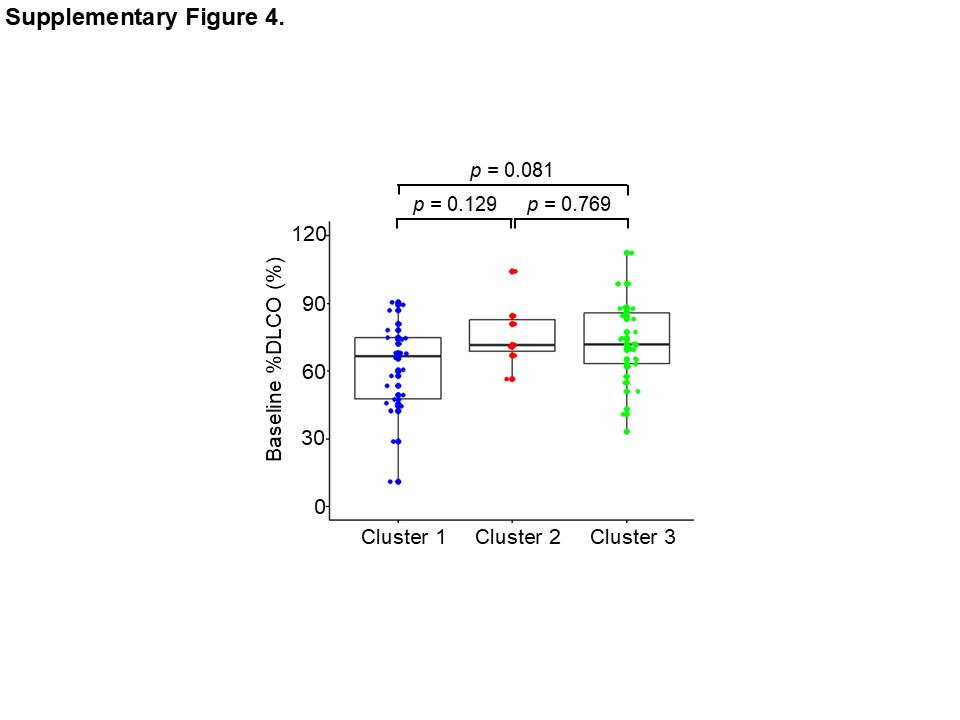

Supplement: Supplementary Figure 4 — Baseline %DLCO in each cluster. Actual baseline %DLCO values for Clusters 1–3 are shown (Total n = 63; Cluster 1, n = 26; Cluster 2, n = 7; Cluster 3, n = 30). [file Image4.jpeg]
